# Cultural Care Practices Provided at Home by the Zenú Indigenous Mothers to their Premature Children and to Those with Low Birth Weight*

**DOI:** 10.17533/udea.iee.v40n2e09

**Published:** 2022-09-19

**Authors:** Yalena Ortiz Anaya, Juan Guillermo Rojas

**Affiliations:** 1 Nurse, Master’s. Professor, Faculty of Nursing, Universidad de Sucre. Sincelejo (Colombia). Email: yalena.ortiz@unisucrevirtual.edu.co. Corresponding author Universidad de Sucre Faculty of Nursing Universidad de Sucre Sincelejo Colombia yalena.ortiz@unisucrevirtual.edu.co; 2 Nurse, PhD. Full Professor, Faculty of Nursing, Universidad de Antioquia, Medellín (Colombia). Email: guillermo.rojas@udea.edu.co Universidad de Antioquia Faculty of Nursing Universidad de Antioquia Medellín Colombia guillermo.rojas@udea.edu.co

**Keywords:** infant, premature, kangaroo-mother care method, indigenous culture, qualitative research., recién nacido prematuro, método madre-canguro, cultura indígena, investigación cualitativa, recém-nascido prematuro, método canguru, mães, pesquisa qualitativa

## Abstract

**Objective.:**

The work’s aim was to comprehend the cultural practices of the care by Zenú indigenous mothers to their newborn premature children and those of low birth weight by implementing the Kangaroo-Mother method at home.

**Methods.:**

Qualitative study of particularistic ethnographic approach, with participation from eight mothers and two key informants trained in the Kangaroo-Mother method, who were interviewed and observed in their homes, in the municipalities of San Andrés de Sotavento, Tuchín, Sampués, and San Antonio de Palmitos from the Departments of Córdoba and Sucre (Colombia), respectively. Ethnographic analysis was performed. The criteria of data saturation and methodological rigor, typical of qualitative research, were applied.

**Results.:**

Eight Zenú indigenous mothers and two key informants from the family participated in the study. The themes emerging were the context, a different experience, adaptations of the Kangaroo-Mother method at home and care practices, protection and healing based on customs and cultural tradition.

**Conclusion.:**

The indigenous mothers provide holistic care to their newborn premature children and those with low birth weight, by integrating the knowledge and practices of the Kangaroo-Mother method and with the ancestral practices of care, protection, and healing characteristic of the context and culture; thus, transcending the use of resources available in the environment.

## Resumen

**Objetivo.:**

Comprender las prácticas culturales del cuidado de madres indígenas Zenú a sus hijos recién nacidos de bajo peso al nacer y prematuros al implementar el Método Madre Canguro en el hogar.

**Métodos.:**

Estudio cualitativo de enfoque etnográfico particularista en el cual participaron ocho madres y dos informantes clave entrenadas en el Método Madre Canguro, quienes fueron entrevistadas y observadas en sus domicilios, en los municipios de San Andrés de Sotavento, Tuchín, Sampués y San Antonio de Palmitos de los Departamentos de Córdoba y Sucre (Colombia), respectivamente. Se realizó análisis etnográfico. Se aplicaron los criterios de saturación de los datos y del rigor metodológico, propios de la investigación cualitativa.

**Resultados.:**

Participaron ocho madres indígenas Zenú y dos familiares informantes claves. Emergieron temas: el contexto, una experiencia diferente, las adaptaciones del método madre canguro en el hogar y las prácticas de cuidado, protección y curación basada en las costumbres y la tradición cultural.

**Conclusión.:**

Las madres indígenas brindan cuidado holístico a sus hijos recién nacidos con bajo peso al nacer y prematuros, al integrar el conocimiento y prácticas del Método Madre Canguro y con las practicas ancestrales de cuidado, protección y curación propias del contexto y la cultura; transcendiendo así el uso de recursos disponibles en el medio.

## Resumo

**Objetivo.:**

Compreender as práticas culturais de cuidado de mães indígenas Zenú para o baixo peso ao nascer e recém-nascidos prematuros na implantação do Método Mãe Canguru no domicílio.

**Métodos.:**

Estudo qualitativo com abordagem etnográfica particularista, no qual participaram oito mães e dois informantes-chave treinados no Método Mãe Canguru, que foram entrevistados e observados em suas residências, nos municípios de San Andrés de Sotavento, Tuchín, Sampués e San Antonio de Palmitos de los Departamentos de Córdoba e Sucre (Colômbia), respectivamente. Foi realizada análise etnográfica. Foram aplicados os critérios de saturação de dados e rigor metodológico, típicos da pesquisa qualitativa.

**Resultados.:**

Participaram oito mães indígenas Zenú e dois familiares informantes-chave. Emergiram temas: o contexto, uma experiência diferente, as adaptações do método mãe canguru em casa e as práticas de cuidado, proteção e cura baseadas em costumes e tradição cultural.

**Conclusão.:**

As mães indígenas prestam cuidados holísticos aos seus recém-nascidos de baixo peso e prematuros, integrando os saberes e práticas do Método Mãe Canguru e com as práticas ancestrais de cuidado, proteção e cura típicas do contexto e da cultura; transcendendo assim o uso dos recursos disponíveis no ambiente

## Introduction

Globally, each year there are 15-million pre-term births 1 and nearly 20-million newborns with low birth weight.^2^ In both cases, a direct relation exists with neonatal mortality, either as the main cause, or as a predictive factor for the suffering of chronic noncommunicable diseases during later stages of life. In addition to the foregoing, low weight occurs with a higher proportion in low- and middle-income countries and in vulnerable populations,^2^ which is why, the 2030 Sustainable Development Goals seek to reduce neonatal mortality by at least 12 neonates for every 1,000 live births.^3^

In Colombia, during the first period of 2021, registered a rate of low birth weight of 98.4 per 1,000 live births; in the departments of Córdoba and Sucre, this indicator reached 91.2, and 101.7 per live births, respectively.^4^ In addition, the rate of pre-term births per every 1,000 live births in the departments of Córdoba and Sucre was 115 and 126 per live births, respectively.^4^ The specific statistical analysis of the ethnic groups, self-recognized as indigenous, evidences a proportion of pre-term newborns of 9.3%.^4^ In this sense, given the importance of ancestral peoples and the behavior of the indicators, it is significant to focus knowledge on the context and circumstances surrounding the experience of caring for premature newborns and newborns with low weight in these populations.

Regarding the problem of low birth weight and prematurity, implementing the Kangaroo-Mother method (KMM) has permitted saving the lives of nearly 450,000 premature babies each year,^5^ given the multiple benefits that impact positively on the humanization and quality of care, on the health of the newborns and those with low birth weight, and on mortality indicators.^6^^-^7 Under this panorama, it is relevant to specify that, the success of its implementation at home depends on the training in the KMM carried out in the hospital,^8^ more so in the indigenous communities, in whom language becomes a barrier to implement the method; hence, as proposed by Raffray *et al.*,^8^ and Siedman,^9^ knowledge of the cultural context of implementing the KMM should be expanded in indigenous communities through ethnographic research that account for these two aspects, especially when no studies exist to unveil the way the experience of applying the KMM takes place in indigenous communities. Thereby, the objective of this research was to comprehend the care practices of a group of Zenú indigenous mothers from Córdoba and Sucre, by describing the experiences in implementing the KMM at home, taking the cultural traits as starting point.

## Methods

A qualitative study was conducted with particularistic ethnographic approach 11 From the database of the Kangaroo Mother Program in a private hospital in the city of Sincelejo, Colombia, the study selected eight indigenous mothers, members of the Zenú community from the municipalities of San Andrés de Sotavento, Tuchín, Sampués, and San Antonio de Palmitos, who were trained in the KMM between 2018 and 2019 and whose newborn children left the institution alive. Access to the field was through two gatekeepers, the coordinating nurse of the neonatal intensive care unit, who made the first contact with the participants and introduced the researcher, and a motorcycle-taxi driver from the zone, who knew the territory and its inhabitants. A preliminary study was carried out, which served to refine the techniques and guide questions. The study employed semi-structured interview and observation techniques, using a guide. Prior to the visits, the dates to meet with the researcher were arranged by telephone.

Once at home, each participant was explained the aim of the research, addressing doubts about such, the informed consent process was conducted, and the study proceeded with interviews and observations. The observations began from the researcher's travel route to the participants' home. The work used 40 h of observation, both in the territory and in the homes of the participants and key informants. The observations were consigned in the field diary. The time employed for the interviews was 11 h, with an average between 45 and 50 min per interview. Each interview began as an informal conversation, formulating open questions to inquire on general aspects and then questions were made about the experiences regarding the care of the newborns at home, such as: Tell me, how has your experience been caring for your premature newborn and/or low birth weight child at home? Each participant was interviewed, with the need to have an additional interview with some participants. The interviews were audio recorded and transcribed by the researcher as soon as possible, to avoid losing relevant information about them and the context in which they were made.

Analysis of the interviews and of the observations was performed in parallel to the collection process, through exhaustive reading of the texts in search for codes that were grouped into categories and themes. To apply the criteria of rigor of qualitative research 12 upon finishing each interview, a recount of the conversation was made to each participant; as well as the return and validation of the final findings. Complementarily, researcher triangulation was conducted (principal researcher, the research tutor, and a nurse researcher expert on ethnography). This view broadened the analytic perspective and comprehension of the experience and context of the phenomenon. For the theoretical linking process, the topics were contrasted with the theoretical aspects of the Health Traditions Model, proposed by Rachel Spector 13 to describe and comprehend care practices and beliefs around the maintenance, protection and restoration of the health of newborns. The data saturation criterion was used, once sufficient, relevant, and redundant information was obtained on each theme analyzed. To safeguard the identities of the participants, these were assigned a pseudonym, preserving the ethical principles of respect, justice and confidentiality. Additionally, the study was authorized by the healthcare institution and was supported by the research committee in the Faculty of Nursing at Universidad de Cartagena (Colombia).

## Results

Eight Zenú indigenous mothers and two key informants participated. The mean age of the eight participating mothers was 25 years, with high-school studies and dedicated mainly to household chores alternated with the elaboration of handcrafted weaving in “caña flecha”. With respect to the premature newborns, the gestational age was between 29 and 36 weeks and hospital stay in the neonatal intensive care unit was 25 days. Participation was also secured from key informants, the grandmother and father of one of the newborns, who were active in the training process and carried out the kangaroo mother strategy at home.

In the KMM implementation and practices at home, four main themes emerged: the context; a different experience; KMM adaptations at home; and care practices, protection and healing based on the customs and cultural tradition. In their reports, the participants describe how to care for premature newborns at home, making the necessary adjustments from the resources available within the context and in accordance with the cultural and ancestral care practices learned within their families.

### Theme 1. The Context

The study context was located in disperse rural areas of the municipalities of San Andrés de Sotavento, Tuchín, Sampués, and San Antonio de Palmitos characterized by difficulties of roadway access and deficient transport means. The physical characteristics of the homes keep common features, like the construction materials “bahareque” (wall of interwoven sticks with reeds and mud), basic spatial distribution, and poor basic sanitation conditions, which together represent a framework of difficulty for the caring for premature newborns, added to the economic difficulties of the participating mothers, who derive their livelihood from economic activities, like small-scale crops, raising domesticated animals, and handicrafts, which represent low income, compared with the requirements for caring for premature newborns.

In relation with the economic conditions of the participating mothers, demarcated between the small-scale domestic economy of cultivating corn, cassava and yams; raising domesticated animals, principally for consumption; and elaboration of handmade weaving to manufacture hats and bracelets; and motorcycle taxi activities or the various trades of their companions.

### Theme 2. A Different Experience

Unlike the experience in the hospital, the mothers manifest feeling freedom, tranquility and confidence of caring for their children at home, as a “different” and positive experience: *It was a totally different experience, but at the same time beautiful, because, well, you are already living here out in the open, you are not there locked up […] how should I say, I had her here at home, I could hold her, lay down with her; it was something quite beautiful (E1P7Orquídea).*

### Theme 3. KMM Adaptations at Home

During the adaptation process of the KMM at home, the participants put into practice the teachings received in the clinic about issues, like feeding the child and the mother, hygiene, and protection measures to care for the newborns: *One of the things I put into practice the most was feeding, that is, primarily on his hygiene, on the part of hygiene and on the medications, and well on hygiene, bathing him, that is, when he was discharged because he was tiny, they told me there that I could not bathe him until he weighed two-thousand five-hundred grams (E10P17Violeta).*

The bath activity as care and hygiene underwent location adaptations at home. Also, inclusion of plants known for their medicinal use plays a protective function complementary to the cultural care actions focused on preventing and healing diseases acquired from the environment, like “cold”: *Because in the clinics there is a lot of air, a lot of air conditioning, they say that babies get cold […] So if, if baths are applied, one comes and makes a guava leaf bath with orange and those things […] Guava leaf works according to them to relax them, to make them drowsy, if they have a cold, the cold goes down (E1P16Orquidea).*

Hand washing with liquid soap, as a hygiene measure learned by the mothers in the hospital, continued being performed at home, as a protection measure against risks of infection, added to the use of masks for people in contact with newborns: *So I said, no!, everyone who is coming over, well there is the liquid soap, they have to wash their arms to here (pointing to the elbow) and to go in there, I bought some facemasks for them to wear, yes, well and that’s how it was done, based on care too (E12P17Crisantemo).*

When the mothers recognized the fragility of their children, they implemented protection isolation measures, like environmental control and limitation of visits against potential diseases; such is the case of contact with smoke from the kitchen and exposure to insects and mosquitos: *My mother always told me when she was cooking, the smoke, she always kept me with her (the baby) in the bedroom, locked the door and always sent me in there with her (the baby) in the room, that way to protect her from the smoke and all that […] Mom always told me that, given that there are mosquitos around here, one always has to use the insect canopy […] I always had to place a cap on the baby’s head, because, always… that they have the... The winds enter the baby through the head (E5P4, 5, 8 Girasol).*

Controlling visits means for the mothers avoiding contact of the children with women who are pregnant, menstruating, or who recently had engaged in sexual intercourse, given that the children can contract traditional diseases recognized by the indigenous culture, as is the case of “pujo” or the “evil eye”: *So these are customs we still have, so everyone would tell me to not let everybody inside because the child will get “pujo”, so they are customs we have here as indigenous (laughter) […] As we Indians commonly call it here (laughter), we have customs, that we say if so and so comes over with the menstrual period or had sexual relations so they will get the baby sick (E1P15,16Orquidea).*

The mother’s consuming foods considered stimulants of breast milk and avoiding consumption of others that affect the health of the newborn, constitute adaptations of the context and a form of their indirect care of the child: *The indigenous, just like me, have beliefs and that, they said, we here said to eat salted meat, that sesame seed, milk with panela, all that I took to get breast milk (E3P9Amapola); Avoiding heavy meals, like too much fat, drinking lots of liquids, soup every day and natural juice to help her and so the girls […] Both your food that you have to take too, also has to be controlled so that you can't affect the girls (E12P12,15 Crisantemo).*

Likewise, the particularities and feeding needs of each child, suppose necessary adjustments to meet this need, which implies even using traditional knowledge and putting them to practice: *So my mom would say, let's make rice chicha, corn chicha, little things like that, well for one, well, for the eldest, because the other was very difficult because everything she made for him, that is, made him have loose stools, his stomach never tolerated it , then we prepared for one, yes and we gave him (E3P22Amapola).*

### Theme 4. Care Practices, Protection and Healing Based on the Customs and Cultural Tradition

In their narratives, the mothers describe “pujo” in the child with symptoms, like abdominal pain and overall discomfort, associated occasionally with fever episodes and which they prevent by limiting visits, as already narrated. They describe the “evil eye” as general discomfort, head fever, cold extremities, and constant crying of the child and which is acquired by strong stares from other people toward the child: *The evil eye, they say comes from other people and who have a bad eye (E3P15Amapola); Because his head is hot and the other parts of his body are cool, only his head gets hot and the eyes, sort of as in tears, that is, from the very headache he has, so well, that is when you realize that is “eye” what he has and that at night does not let you sleep, because he gets startled so suddenly and starts crying (E10P21Violeta).*

As healing measure, the mothers describe curative actions to counteract “the bad energy” of the person causing the condition in the newborn, as illustrated in the following account: *That when a person gives you the evil eye, well, there is the custom that you go to that person and take a piece of clothing, that is, that the person has worn… you rinse with that… you place it, let's say, on the trail or on the royal road and make a cross, the cross is for when people pass by there, they take away that bad energy, they take it until it is pushed away (E12P16Crisantemo).*

For its part, "the vision" or "sorcerer's vision" is described by the participants as a disease caused by taking the child out of the home without an amulet, without having been baptized or taking them at night through solitary paths in which sorcerers or spirits could potentially appear. Given this, the mothers take actions to protect the newborns or counteract the condition, as shown by the following testimony: *When at night I would dress the baby with red garments, but inside out […] Because when they are small there are many visions they see and that is very dangerous; as they say, those sorcerers come out, when the children are just born, they like to play with the babies (E5P5,6Girasol).*

In the testimonies by the mothers complementary actions emerged of protective and curative nature and to counteract the “bad energies” or “bad spirits” that cause the aforementioned diseases, evoking ancestral practices and rituals typical of the culture, like the use of “amulets” and the “prayers”: *We had to tie one on, this one, there is a little animal that is like a little cricket and it has some pebbles, some little things, so they tie them to their hand, that is done, it has to be done in odd number…then you make the bracelet and tie it to the baby’s left hand so that they don't even have vision…evil eye or they get vision on the path and that is done (E3P15Amapola).*

As a theoretical linking strategy and to advance in comprehending the cultural practices, the study used the holistic conception of Spector’s Health Traditions Model,^14^ according to which balance is required in body, mind and spirit, family, community and natural forces; reached through maintaining and defending the traditional beliefs and practices that persist in people who know and live according to the traditions of their ethnocultural or religious heritage.^15^Table 1 gathers the data derived from the observations and interviews, within the framework proposed by the author.

**Table 1:** Personal perspective in Health Traditions and implementation of the KMM in Zenú indigenous people from Córdoba and Sucre

	Physical	Mental	Spiritual
Maintenance of Health	Use of red-colored garments to avoid sorcerers and caps to keep the cold from entering the child. Consume food to improve the production of maternal milk. Suspend routine household and artisanal activities.	Support from family members in implementing the KMM that provides security, tranquility and confidence to the mothers at home.	Spirituality seen in prayers to improve the mother’s and child’s health, asking for help to cope with the experience and seeing the child as a miracle.
Protection of Health	Use of red-colored garments in children. Avoid foods that cause illnesses to the mothers.	Prevention from visits by women who are menstruating, who have had intercourse and avoid taking the children out to dark and solitary places, such as dams and wells that cause “Evil eye” “Pujo” and Visions Family activities: protection baths.	Use of amulets and other symbolic objects, red-colored garments to prevent the "evil eye" or to defray other types of damage; “Mate” in hands and/or feet to cross children and avoid endemic diseases.
Health restoration	Baths with medicinal plants to heal the children from illnesses like the flu.	Use of medicinal baths for cleansing and healing with traditional medicinal plants from the region, others such as azulene water, water product of the rinsing of clothes, cow's milk.	Sanctify the children with traditional healers of the culture, bathe the children with the garments of the person who caused them the “evil eye”, bathe them and throw away the water making the sign of the cross.

## Discussion

The experience of caring for newborns at home is frame-worked by learning and knowledge acquired during the KMM training and the adequate practice, which agrees with the proposal by Abanto *et al*.,^16^ inasmuch as certain confidence is generated when applying what has been learned at home, and guides the adequacy of care with the resources available in the context. Although the mothers indicate that the experience of homecare is different by virtue of these adaptations, no negative feelings or inability to care for newborns are generated, which can be determined by accompaniment from other members of the family group; an aspect described by other authors as a positive factor in caring for the premature child at home.^17^^-^18 Additionally, there is the satisfaction of having their children at home, as identified by Osorio *et al.*^19^ These practices agree with the knowledge the mothers acquire during the KMM training KMM indicated by Abanto *et al*.,^((^16 in which - with a higher level of knowledge of homecare in the dimensions of security, protection, comfort, and feeding - said mothers will conduct better practices on their newborns, also contrasting with that reported by others, like Castiblanco,^20^ with respect to basic care of premature newborns, recognizing warning signs, administration of medications, vaccine applications, and follow up of medical and nursing indications, as a distinctive feature of maternal basic care and as measures to provide comfort and protection to premature children,^21^ reflecting among the participants on the conservation and control of the environment, maintenance of body heat, application of hygiene measures, baths, and their adjustments for the protection of the newborns. Further, it accounts for other experiences of the indigenous in hospital to satisfy their basic needs and overcome their limitations through adaptation and learning 22 This synergy between knowledge acquired during KMM training and traditional knowledge, such as adjustments in feeding the mother to stimulate maternal breastfeeding and prevent diseases in the newborn, guides the care of the dyad and tributes positively on the child’s and mother’s health.^23^

Comprehending the experience and applying some elements from the Health Traditions model by Rachel Spector^13^, guided by the logical and comprehensive analysis of the ethnographic approach of the research, grant relevance to the context and to cultural aspects, emerging in a flourishing way the care of the premature newborn and with low birth weight based on the beliefs, ancestral practices and myths that promote the protection, treatment and healing of the newborn, also described by Banda *et al.,*^24^ and the adaptations conducted on the care environment from the knowledge gained and experiences of the KMM training. Besides the basic care aimed at satisfying the basic needs of the children, care by the mothers seeks to protect and heal the child from diseases known culturally among the Zenú as “pujo”, the “evil eye”, and the “visions” and some studied by authors^25^^-^26 in other cultures that describe them with similar causes, manifestations and forms of healing guided by own resources of ancestral knowledge, such as the use of amulets or the use of garments, baths with of medicinal use, traditional healing prayers, and use of amulets, like protection collars or bracelets.^27^ It is important to highlight that the study observed no unfavorable cultural practices or practices that endangered the newborn.

The principal limitations of this study had to do with the difficulties of geographic accessibility in disperse rural zones, place of residence of some of the participants, as well as the telephone communication with the participants due to poor coverage by mobile operators in the zone or no access to their own telephone number.

In conclusion, this study has it that comprehending the KMM practice at home is conditioned with the context of the environment, geographic and socio-economic conditions, cultural heritage, and family support of each participant, which have a strong cultural component. Applying the KMM at home for the group of participants is holistic and congruent care, which incorporate new knowledge from the learning acquired during the KMM training and el traditional care based on el ancestral knowledge learned within the indigenous culture. Thus, the KMM dialogues with the traditional knowledge and permeates culture through the nursing professional’s teaching role in the neonatal intensive care units. This cultural approach is a starting point to promote cultural competence and adaptation of care to the conditions of the people, taking culture as referent, particularly in a multicultural country, like Colombia, where - according with the results - intense work must be done in the incorporation of substantial changes in formation scenarios and in professional practice scenarios to improve cultural care by nurses. Moreover, it constitutes the input for the construction and consolidation of public policies of intercultural and ethnic care with territorial approach.
